# Inducible and Targeted Deletion of the ERK5 MAP Kinase in Adult Neurogenic Regions Impairs Adult Neurogenesis in the Olfactory Bulb and Several Forms of Olfactory Behavior

**DOI:** 10.1371/journal.pone.0049622

**Published:** 2012-11-21

**Authors:** Yung-Wei Pan, Chay T. Kuo, Daniel R. Storm, Zhengui Xia

**Affiliations:** 1 Graduate Program in Molecular and Cellular Biology, University of Washington, Seattle, Washington, United States of America; 2 Departments of Cell Biology and Pediatrics, Duke University Medical Center, Durham, North Carolina, United States of America; 3 Department of Pharmacology, University of Washington, Seattle, Washington, United States of America; 4 Toxicology Program in the Department of Environmental and Occupational Health Sciences, University of Washington, Seattle, Washington, United States of America; Duke University, United States of America

## Abstract

Although adult-born neurons in the subventricular zone (SVZ) and olfactory bulb (OB) have been extensively characterized at the cellular level, their functional impact on olfactory behavior is still highly controversial with many conflicting results reported in the literature. Furthermore, signaling mechanisms regulating adult SVZ/OB neurogenesis are not well defined. Here we report that inducible and targeted deletion of *erk5*, a MAP kinase selectively expressed in the adult neurogenic regions of the adult brain, impairs adult neurogenesis in the SVZ and OB of transgenic mice. Although *erk5* deletion had no effect on olfactory discrimination among discrete odorants in the habituation/dishabituation assay, it reduced short-term olfactory memory as well as detection sensitivity to odorants and pheromones including those evoking aggression and fear. Furthermore, these mice show impaired acquisition of odor-cued associative olfactory learning, a novel phenotype that had not been previously linked to adult neurogenesis. These data suggest that ERK5 MAP kinase is a critical kinase signaling pathway regulating adult neurogenesis in the SVZ/OB, and provide strong evidence supporting a functional role for adult neurogenesis in several distinct forms of olfactory behavior.

## Introduction

Humans and other mammals detect and distinguish between thousands of different odorants. Defects in olfaction cause loss of appetite and poor nutrition, particularly with older individuals. For example, the average human loses a significant proportion of their olfaction as they age, and olfactory dysfunction is associated with several neurodegenerative diseases including Alzheimer’s and Parkinson’s disease [1]. Recent studies led to the exciting discovery of ongoing adult neurogenesis in the subventricular zone (SVZ) along the lateral ventricles in the adult brains of mammals [2]–[5]. Newly generated neuronal precursors in the SVZ migrate along the rostral migratory stream (RMS) to the core of the olfactory bulb (OB) where they begin radial migration and differentiation into inhibitory interneurons [6], [7]. Although these neurons have been extensively characterized at the cellular level, their functional impact on olfactory behavior is still an open question [6], [8]–[11]. Furthermore, signaling mechanisms regulating adult SVZ/OB neurogenesis are not fully defined. Consequently, it is critical to elucidate signaling mechanisms regulating adult SVZ/OB neurogenesis and to generate definitive evidence concerning the role of adult neurogenesis in olfactory behaviors.

ERK5 is a member of the MAP kinase super family of proteins that include ERK1/2 [12], [13]. We recently discovered that although its expression in the adult brain is extremely low, ERK5 is prominently expressed along the neurogenic SVZ-RMS-core of the OB pathway. This striking pattern of expression suggests a unique and important role for ERK5 signaling in adult SVZ/OB neurogenesis. We have generated an ERK5 inducible and conditional knock out (icKO) mouse that allows us to delete the *erk5* gene specifically in neurogenic regions of the adult brain [14]. Here, we report that ERK5 icKO mice are impaired in adult neurogenesis in the SVZ/OB and in several forms of olfactory behavior.

## Materials and Methods

### Ethics Statement

All animals used in this study were approved by the University of Washington Institutional Animal Care and Use Committee. Experimental conditions and procedures were performed with direct approval under protocol 3041-04.

### Animals

Nestin-CreER™/ERK5^loxP/loxP^ and ERK5^loxP/loxP^ transgenic animals have been previously described [14], [15]. Littermates were handled identically and housed under standard conditions (12 h light/dark cycle) with food and water provided *ad libitum* except where indicated.

### Reagents

The following primary antibodies were used for immunohistochemistry: rat anti-BrdU (1∶500, AbD Serotec), mouse anti-NeuN (1∶500, Millipore), and polyclonal goat anti-doublecortin (DCX, 1∶200, Santa Cruz Biotech. Inc.). Affinity-purified polyclonal ERK5 antibody (1∶500) was described previously [14], [16]. The following odorants were used: citralva (International Flavors & Fragrances, Inc.), isoamyl acetate (IAA, Sigma), ethyl vanillin (SAFC Global), S-terpinen-4-ol (Sigma), acetophenone (Sigma), 1-octanol (Sigma), 2-heptanone (Sigma), farnesene (Wako USA), 2,3,5-trimethyl-3-thiazoline (TMT, Phero Tech).

### Tamoxifen and BrdU Administration

**Figure 1:**
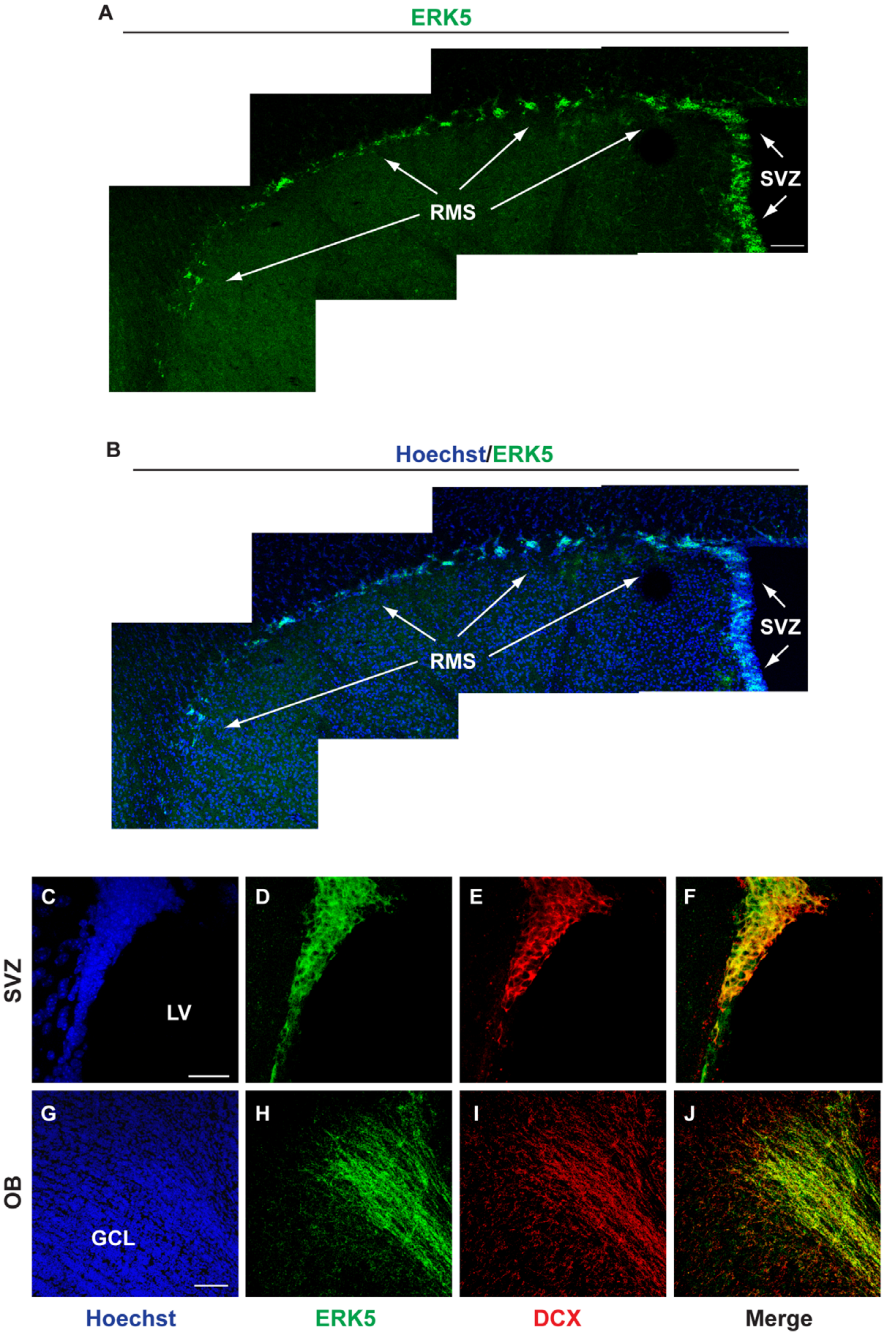
ERK5 is expressed along the entire SVZ/RMS/OB axis. A, B) ERK5 protein (green) was identified by IHC along the SVZ and RMS of adult mouse brain. Hoechst staining (blue) was used to identify all cell nuclei. Scale bar in A represents 100 µm and applies to B. C–J) ERK5 co-localizes with DCX^+^ cells (red) along the lateral ventricles (LV) and in the center of the granular cell layer (GCL) of the OB where adult born neurons exit the RMS. Scale bar in C represents 25 µm and applies to D–F, while scale bar in G represents 100 µm and applies to H–J. Three individual mouse brains were analyzed for ERK5 expression along the SVZ/RMS/OB axis.

**Figure 2:**
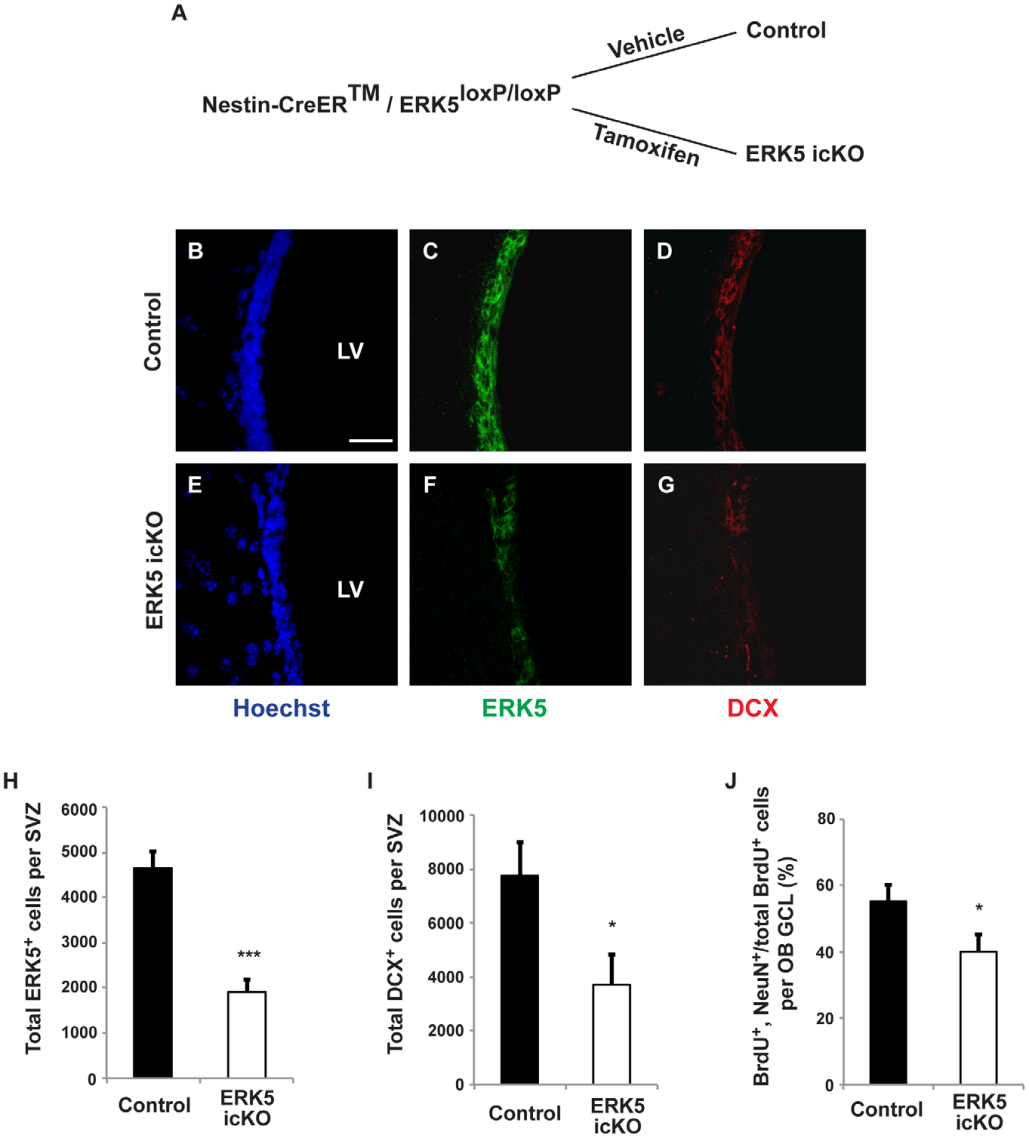
Inducible and conditional knock out of *erk5* significantly reduces ERK5 protein expression along the SVZ, the number of DCX^+^ cells in the SVZ, and the number of adult-born neurons in the OB. A) Schematic illustration of the experimental design. B–G) Representative immunostaining photomicrographs of ERK5 (green) and DCX (red) in vehicle control-treated mice (B–D) as well as tamoxifen-treated ERK5 icKO mice (E–G). Scale bar in B represents 25 µm and applies to C–G. H) Quantification of total ERK5^+^ cells along the SVZ. I) Quantification of total DCX^+^ cells along the SVZ. J) The deletion of *erk5* decreases the total number of adult-born neurons (BrdU and NeuN double-positive cells among total BrdU^+^ population) in the granule cell layer of the OB 3 weeks after BrdU injection. n = 5 individual mouse brains and olfactory bulbs per treatment condition.

**Figure 3:**
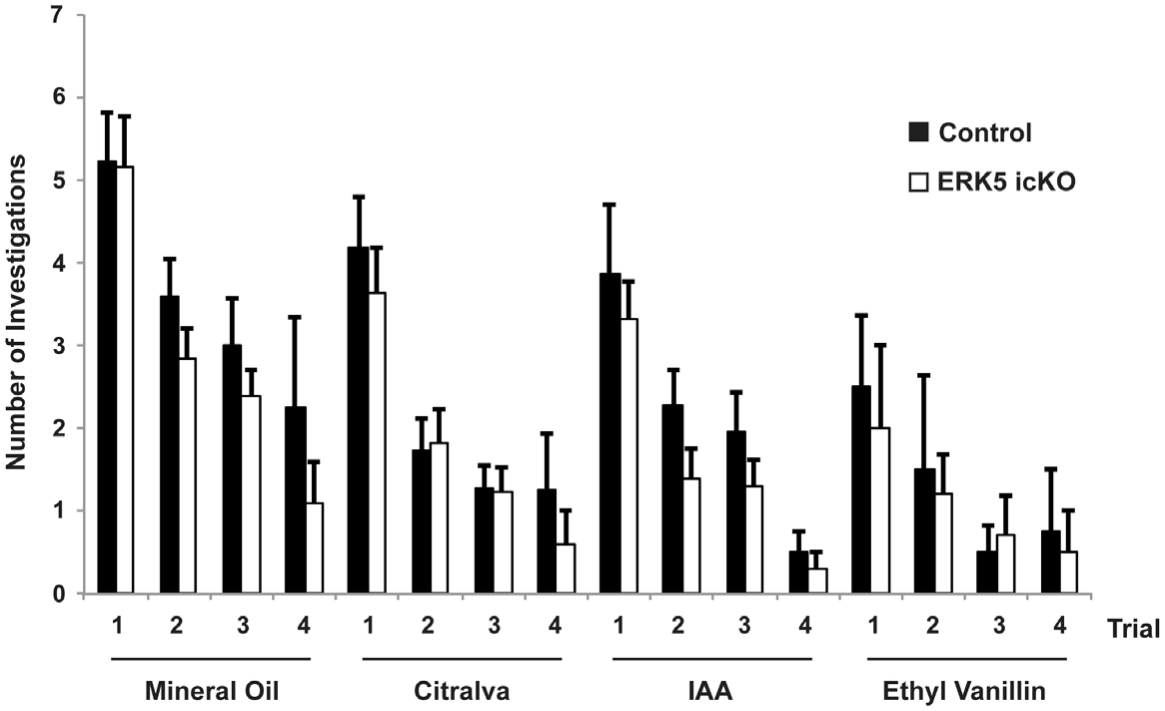
ERK5 icKO mice have normal olfactory detection to discrete odorants in a habituation/dishabituation assay. Naïve, adult mice were pre-trained with four presentations of mineral oil-soaked cotton swabs, then exposed to three structurally different odorants, citralva, isoamyl acetate (IAA), and ethyl vanillin with 4 trials per odorant. Step-wise decrease in the number of investigation during sequential presentations of the same odor followed by renewed interest in investigation of the first presentation of a new odorant suggests normal olfactory habituation/dishabituation behavior. n ≥16 mice per treatment group from 2 independent experiments.

**Figure 4:**
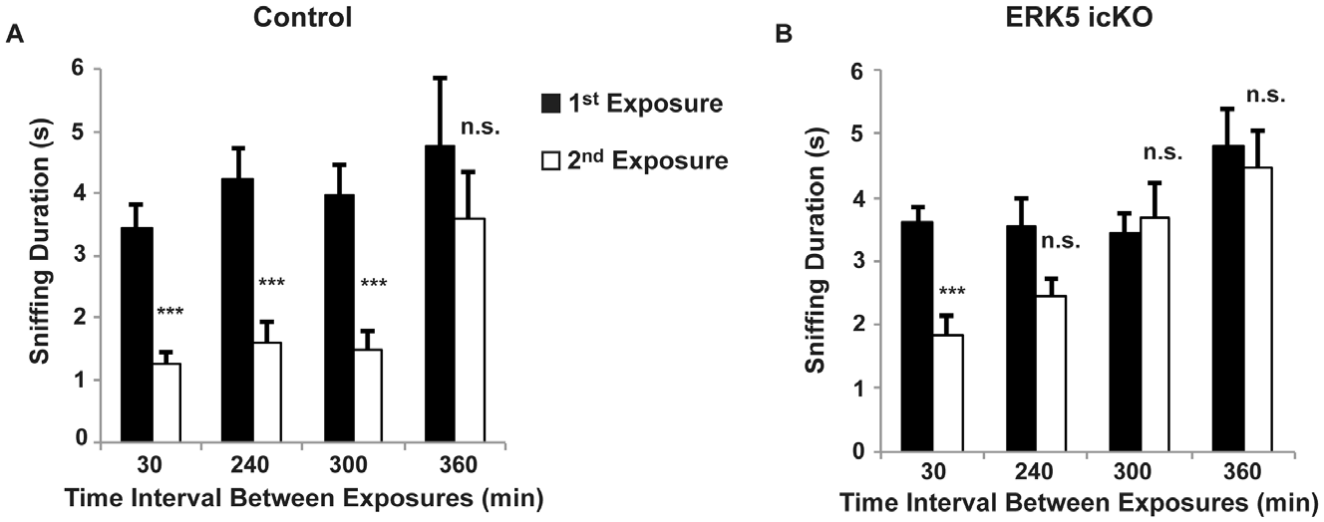
ERK5 icKO mice are deficient in olfactory short-term memory. Mice were presented with cotton swabs laced with the same odorant twice at the indicated time intervals. A different odor was used for each interval time point. The sniffing duration of the cotton swab was recorded for both exposure sessions. A decrease in investigation during the second exposure of the same odorant is suggestive of olfactory memory to the odorant. A) Short-term olfactory memory of control mice. B) Short-term olfactory memory of ERK5 icKO mice. n ≥7 mice per treatment group.

**Figure 5:**
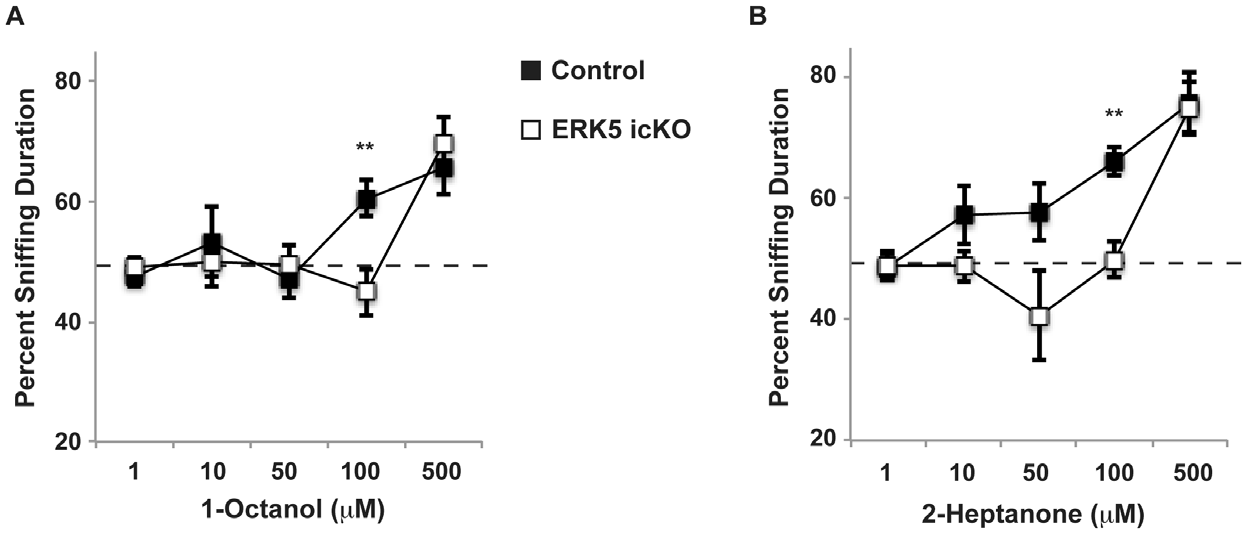
Reduced detection sensitivity to 1-octanol and 2-heptanone in ERK5 icKO mice. ERK5 icKO and control mice were presented with 2 cotton swabs, one laced with vehicle control and the other with increasing concentrations of 1-octanol or 2-heptanone. An above 50% sniffing duration (above chance) indicates detection of 1-octanol or 2-heptanone at that specific concentration. A) Dose-response of detection threshold to 1-octanol, a chemically defined odorant. B) Dose-response of detection threshold to 2-heptanone, a chemically defined mouse pheromone. n ≥14 mice per treatment group.

**Figure 6:**
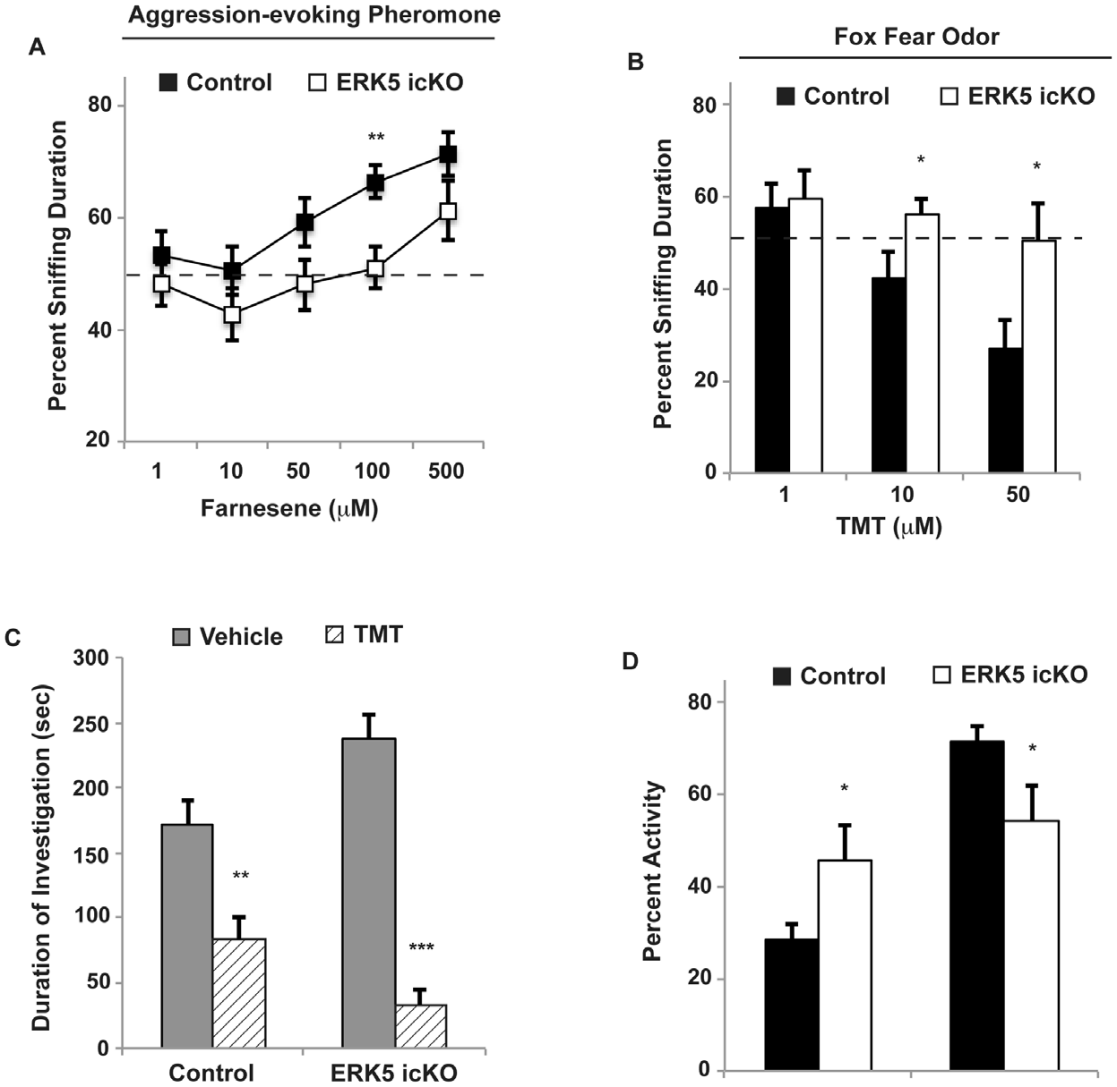
ERK5 icKO mice have reduced detection sensitivity to aggression-evoking and fear-inducing pheromones, but are not impaired in innate fear response *per se*. A) Dose-response of detection threshold to farnesene, a chemically defined, aggression-evoking mouse pheromone. ERK5 icKO and control mice were presented with 2 cotton swabs, one laced with vehicle control and the other with increasing concentrations of farnesene. An above 50% sniffing duration (above chance) indicates detection of farnesene. B) Dose-response detection to TMT, a fear-evoking odor found in fox feces. ERK5 icKO and control mice were presented with 2 cotton swabs, one laced with vehicle control and the other with increasing concentrations of TMT. Because TMT should elicit avoidance behavior when mice detect it under normal conditions, a below 50% sniffing duration (less than chance) indicates detection of TMT. C) ERK5 icKO and control mice were subjected to the innate fear assay toward TMT using a 3-chamber apparatus. The duration of investigation toward the chamber containing filter paper laced with TMT (5 mM) or vehicle control was quantified. Avoidance behavior toward the TMT chamber relative to the vehicle control chamber is a measure of TMT detection and innate fear. D) Quantification of percent time mice spent freezing, another measurement of innate fear, during the innate fear assay toward TMT using the 3-chamber apparatus. n ≥7 mice per treatment group.

**Figure 7:**
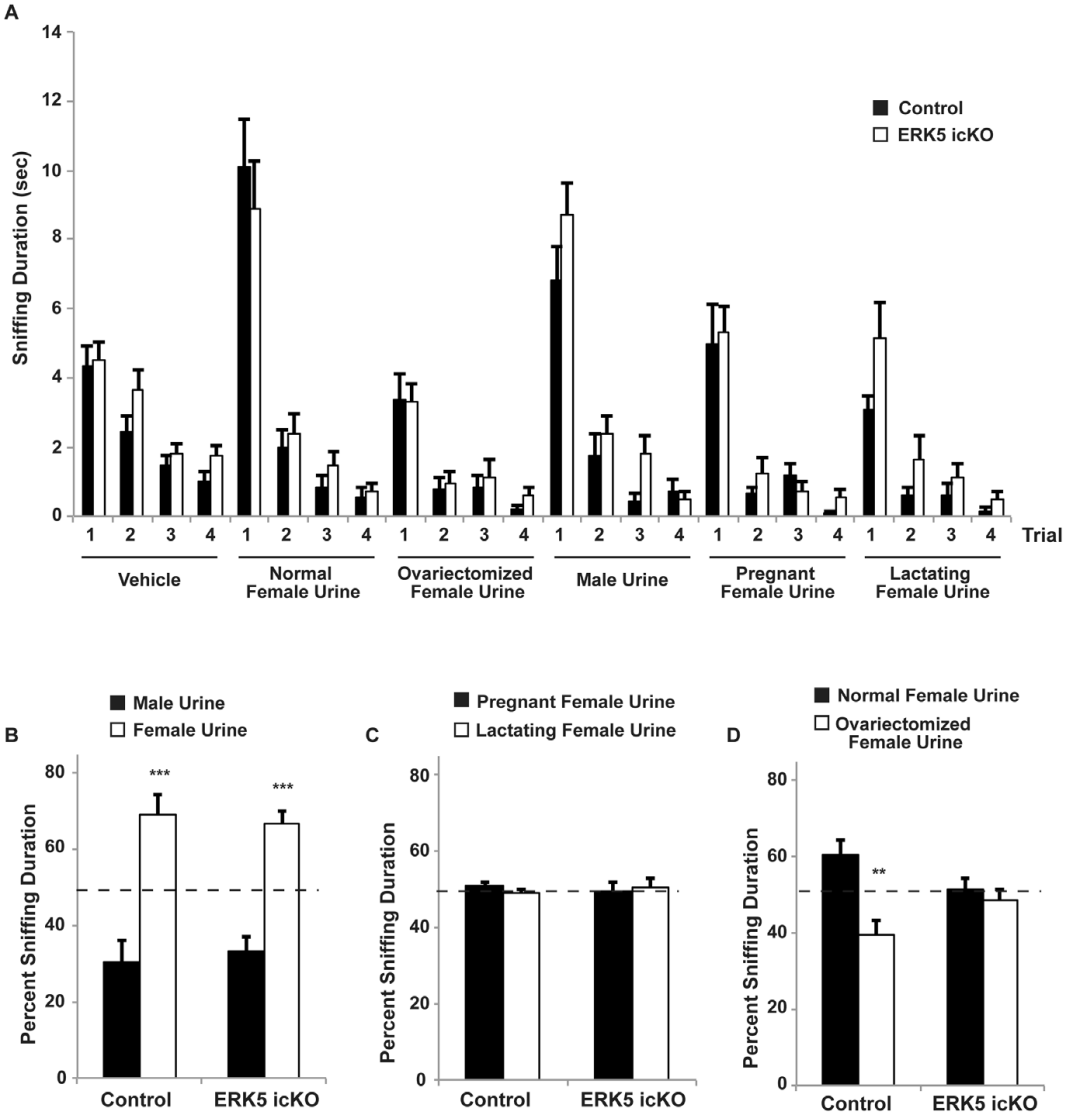
Unlike control mice, ERK5 icKO mice do not prefer mouse urine from normal females to that from ovariectomized females. A) Male ERK5 icKO and control mice were tested for their ability to discriminate between various mouse urine samples using the habituation/dishabituation assay. The duration of sniffing of the cotton swab was quantified. Both groups of mice were able to discriminate between urine samples over the course of 4 sequential presentations. B) Urine preference test between female mouse urine vs. male mouse urine, presented simultaneously with cotton swabs laced with female or male mouse urine. The duration of sniffing toward each cotton swab was quantified. Control and ERK5 icKO mice (both male) showed a significant preference sniffing towards female mouse urine vs. male mouse urine. C) Urine preference test between pregnant and lactating female mouse urine. D) Urine preference test between ovariectomized female mouse urine and age-matched, normal adult female mouse urine. Control mice preferred sniffing normal adult female mouse urine while ERK5 icKO mice showed no preference toward either. n ≥7 mice per treatment group.

**Figure 8:**
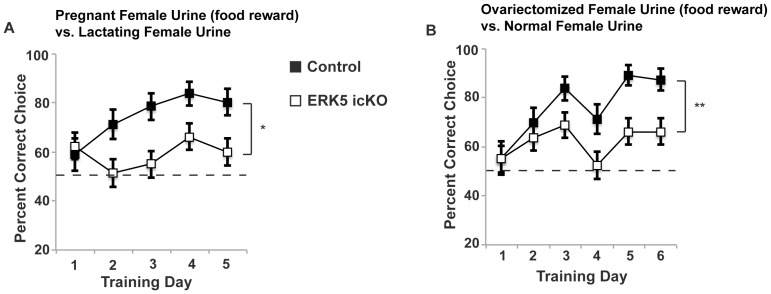
ERK5 icKO mice are impaired in the acquisition of a sand digging-based, **odor-cued associative learning.** A) Control, but not ERK5 icKO mice learned to associate a food reward with pregnant female mouse over a 5-day training course. B) ERK5 icKO mice did not learn as well as control mice to associate a food reward with ovariectomized female mouse urine over a 6-day training course. n ≥7 mice per treatment group.

Adult (8–10 wk old) male wild type mice not treated with tamoxifen nor BrdU were used to determine basal levels of ERK5 expression as shown in Figure 1. For data in all other figures, adult male mice were dosed daily with 200 mg/kg of freshly dissolved tamoxifen (Sigma) in 2% glacial acetic acid in corn oil solution (Sigma) to activate Cre-mediated recombination. Mice were administered tamoxifen either once per day for 7 d for 1 cycle for data in Figure 2 (cellular studies) or once per day for 4 d in each cycle for 3 cycles, with a 2-week inter-cycle interval for Figures 3–8 (behavior studies). BrdU (Sigma) was administered at a dose of 100 mg/kg by intraperitoneal injection 5 times (every 2 h for 10 h) in one day followed by sacrifice 3 weeks later to identify BrdU-retaining, adult-born cells.

### Immunohistochemistry (IHC)

Brains from mice were harvested following intracardial perfusion and stored in −80°C until IHC processing as described [14], [15]. IHC was performed on 20 µm thick coronal olfactory bulb sections and either 30 µm thick coronal or 20 µm thick sagittal brain sections using a free-floating antibody staining method as described [15].

### Confocal Imaging and Analysis

Images were captured with an Olympus Fluoview-1000 laser scanning confocal microscope with numerical aperture (NA) 1.3, 40X oil immersion lens. Optical Z-sections (1 µm) were collected and processed using ImageJ (NIH). Images were uniformly adjusted for color, contrast, and brightness using Adobe Photoshop CS4 (Adobe Systems, Inc.).

### Quantification of Immunostained Cells

One in every 8 coronal serial brain sections containing the SVZ or 1 in every 4 coronal serial olfactory bulb sections was immunostained with the indicated markers. Immunopositive cells were quantified as described [15], using a modified unbiased stereology technique by confocal microscopy with the experimenter blind to treatment conditions [17]–[19]. Hoechst staining of the nuclei was used to confirm corresponding marker expression associated with a specific cell nucleus. Resulting cell numbers were scaled relative to the number of sections per SVZ or olfactory bulb and reported as an estimated total number of cells per region. Approximately 6–10 sections were used for quantification per region per brain. For co-localization analysis, greater than 50 immunopositive cells were randomly selected and analyzed using confocal microscopy for the presence of overlapping fluorescent signal across all planes in an optical Z-series of a single cell. Only when overlapping signal was found in all planes was a cell considered to be double positive.

### Behavior Assays

Male mice were used for behavior assays. Mice were individually housed and handled at least 4 d before olfactory behavior assays and remained singly caged throughout all olfaction assays. With the exception of the TMT-based innate fear assay, all other olfactory behavior assays were conducted in mouse home cages. For all cotton-tip based behavior assays, cotton swabs dipped in vehicle control (mineral oil or water) or odorant solution were suspended from the wire top of the animal’s home cage with the cotton tips 8 cm above the cage floor. The duration of animals’ sniffing of the cotton swab was recorded. The sniffing was defined as animals’ noses approaching to and within 1 cm distance to the swabs. The animal does not have to physically touch it. Odorants and corresponding concentrations used in various olfaction tests are listed in Table 1.

### Olfactory Habituation/Dishabituation Test with Chemical Odorants

This was conducted as described [20]. Briefly, naïve animals were pre-trained with mineral oil-laced cotton swabs for four presentations (60 s each, 2 min intervals) to ensure that subsequent exposure to an odorant-laced cotton swab did not elicit a response due to object novelty. The odor habituation/dishabituation test was then performed by presenting citralva, IAA, and ethyl vanillin sequentially, with four presentations for each odorant (60 s each presentation, 2 min intervals). A significant decrease in the number of investigations during subsequent presentations of the same odorant indicates odor recognition and habituation. An increase in investigation of a new odorant indicates dishabituation.

### Olfactory Habituation/Dishabituation Test with Diluted Mouse Urine

This was conducted as described above using diluted (1∶50) normal female mouse urine, ovariectomized female mouse urine, male mouse urine, pregnant female mouse urine, and lactating female mouse urine, sequentially, with four presentations of each urine sample (60 s each presentation, 2 min intervals). A significant decrease in total investigation time during subsequent presentations of the same urine sample indicates recognition and habituation. An increase in investigation of the next, new urine sample indicates dishabituation.

### Olfactory Short-term Memory

This was performed as described with modifications [21]. Mice were presented with a cotton swab laced with the same odorant during two different 5 min sessions separated by 30, 240, 300, or 360 min intervals. Odorant detection and investigation of the cotton swab was recorded for each 5 min session. A different odor was used for each interval time point, but only one time interval was tested on each day for each mouse to avoid cross interference of olfactory detection and memory. A significant decrease in total investigation time of the cotton swab during the 2^nd^ presentation of the odorant suggests olfactory memory for the 1^st^ presentation of the same odorant [21]–[23].

### Odor Detection Threshold

This was performed as described with modifications [21]. Mice were presented with 2 cotton swabs simultaneously; one laced with vehicle control and the other laced with a specific odorant or pheromone as indicated. The relative location of the two cotton swabs was randomly switched between presentations to avoid spatial learning. One session was performed per day per concentration, with increasing concentrations each day. Each session lasted 3 min and total investigation time of each cotton swab was recorded during the entire 3 min session. Data are presented as the mean ratio between the time spent investigating the odor and the total sniffing of both cotton swabs (percent sniffing duration). A 50% sniffing duration indicates no detection of the odorant because the animals spent an equal amount of time investigating both cotton swabs. Sniffing durations greater than 50% of the time indicates that animals detected the odorant.

To measure detection sensitivity for TMT, the above assay was performed with slight modifications. Since TMT is a known fear-inducing odor component of fox feces [24], we mixed it with odorants that mice are not averse to (1 mM 2-heptanone, 1-octanol, or S-terpinen-4-ol). Mice were then presented with 2 cotton swabs as above, one laced with just the odorant and the other laced with the odorant plus increasing concentrations of TMT. When mice detect TMT, they avoided this cotton swab; thus a sniffing duration less than 50% indicates that the animal detected the fear odor.

### TMT-based Innate Fear Assay

This was conducted in a 3-chamber testing apparatus as described [25], [26]. Briefly, the apparatus (60 cm×22 cm×12 cm) consisted of three 20 cm×22 cm×12 cm partitions. Dividers between chambers had a small opening to allow mice to cross freely between the three chambers. Mice were placed in the middle chamber and a small plastic dish was placed at the far end of each of the other two chambers; mice were allowed to habituate in the apparatus for 15 min. Following habituation, the two plastic dishes were retrieved and a filter paper (2 cm×2 cm) containing 5 µL of water or 5 mM TMT was placed into each of the two dishes in a randomized manner between mice. The dishes were then placed back into the apparatus at their original location; the duration of investigation to each chamber and freezing behavior were recorded during a 5 min test session with the experimenter blind to treatment conditions. Avoidance of the TMT chamber and increased freezing behavior are indicative of innate fear responses.

### Olfactory Preference

Mice were presented simultaneously with two cotton swabs laced with undiluted mouse urine (10 µL). Mouse urine was collected from group-housed mice (n = 4–5 per cage) and pooled over a period of 2 weeks to minimize day-to-day fluctuations in basal pheromone and urine amount. Total investigation time of each cotton swab was recorded during a 2 min session. Cotton swab investigation is defined as the nose of the animal approaching to and within 1 cm from the cotton swab. The animal does not have to physically touch it. Data presented are percent sniffing duration for each odorant or urine relative to the total sniffing of both cotton swabs. A 50% sniffing duration indicates no preference for either urine.

### Sand-digging Based Odor-cued Associative Olfactory Learning

Following olfactory preference assay, mice were food restricted to maintain 85–90% of free-feeding body weight for 4–5 d prior to the beginning and throughout the entirety of associative olfactory discrimination assay, which was performed as previously described using the same apparatus, pre-training, and training protocols [20]. Briefly, after mice learned to associate sand digging with a food reward at the bottom of the dish, mice were presented with 2 sand dishes in each trial, for 4 trials per block, and 2 blocks per day. One dish contained 100 µL of a urine sample associated with a food reward, while the other contained 100 µL of another urine sample (both urine samples were diluted 1∶50) without food reward. To avoid spatial learning, the two dishes were placed on either the left or right side randomly as long as each dish was placed on each side twice per block but no more than three consecutive times in each day. Scoring for correct or incorrect choice was based entirely on the animal’s first dig, either in the urine dish with the food reward (correct) or in the urine dish without the food reward (incorrect). Mice that dug in the incorrect dish were not allowed to self-correct. Two pairs of urine samples were used in this assay: 1) ovariectomized (enforced with food reward) vs. normal female urine; 2) pregnant (enforced with food reward) vs. lactating female urine.

### Statistical Analysis

Repeated measures ANOVA was used to analyze data in Figures 3, 7A, and 8. Student’s *t-*test was used to analyze all remaining data. Data represent mean ± standard error of means (s.e.m.). *, p≤0.05; **, p≤0.01; ***, p<0.001; n.s., not statistically significant (p≥0.05).

## Results

### ERK5 is Expressed Along the SVZ-RMS-OB Axis in Adult Mice

Brain sections from adult mice were processed for ERK5 immunohistochemistry using an affinity-purified polyclonal ERK5 antibody. Although ERK5 protein is not expressed in most regions of the adult mouse brain [15], ERK5 expression was prominent along the SVZ, RMS, and in the center of the granular cell layer of the OB where adult born neurons exit the RMS (Fig. 1). Additionally, we performed co-labeling of ERK5 and doublecortin (DCX), a marker for transiently amplifying progenitors/newborn neurons, and found a high degree of co-labeling in both the SVZ and OB (Fig. 1C–J). This unique expression pattern of ERK5 suggests that ERK5 plays a role in regulating adult SVZ/OB neurogenesis.

### Targeted Deletion of *erk5* Reduces Adult SVZ/OB Neurogenesis

Using a Nestin-CreER™/ERK5^loxP/loxP^ transgenic mouse strain [14], we conditionally deleted *erk5* in Nestin-expressing neural stem cells along both the subgranular zone (SGZ) of the dentate gryus [14] and SVZ (Fig. 2) in adult animals upon tamoxifen treatment (ERK5 icKO mice). Three weeks after a 7-day tamoxifen treatment paradigm, the total number of ERK5^+^ cells was reduced by 60% in the SVZ of ERK5 icKO mice compared to vehicle control-treated littermates (control) (Fig. 2A–H; *t*-test, *p*<0.001). This was accompanied by a 50% reduction in the total number of DCX^+^ cells in the SVZ (Fig. 2I; *t*-test, *p* = 0.03). To determine if the reduction of ERK5^+^ and total DCX^+^ cells in the SVZ had any impact on the number of adult-born neurons in the OB, we dosed animals with BrdU to label adult born, BrdU-retaining cells. Animals were sacrificed 3 weeks later; OB sections were immunostained for BrdU and NeuN, a marker expressed in mature neurons of the granular cell layer of the OB. There was a statistically significant reduction in the percentage of BrdU and NeuN double-positive cells among all BrdU^+^ cells in the granule cell layer of the OB (Fig. 2J; *t*-test, *p* = 0.05), indicating a reduction in the total number of adult-born, mature neurons during this time window (3 weeks after BrdU labeling). These data suggest that ERK5 plays a role in regulating adult SVZ/OB neurogenesis *in vivo*.

### ERK5 icKO Mice can Detect Discrete Odorants in a Habituation/Dishabituation Assay but Show Reduced Olfactory Short-term Memory

To investigate the functional impact of conditional *erk5* deletion in ERK5 icKO mice on olfactory behavior, we first tested animals for an odor habituation/dishabituation assay using three distinct odorants: citralva, isoamyl acetate (IAA), and ethyl vanillin. This assay is commonly used to detect overt odor detection deficits in mice [27]. ERK5 icKO mice showed normal habituation/dishabituation and were indistinguishable from control mice (Fig. 3; ANOVA_mineral oil_, *p* = 0.06; ANOVA_citralva_, *p* = 0.22; ANOVA_IAA_, *p* = 0.06; ANOVA_ethyl vanillin_, *p* = 0.25).

When tested for short-term olfactory memory, both control and ERK5 icKO mice spent significantly less time sniffing the odor-laced cotton swab during the second exposure than the first exposure when the same odorant was presented 30 min apart (Fig. 4; *t*-test_Control_, *p*<0.001; *t*-test_ERK5 icKO_, *p*<0.001). There was no statistically significant difference between control and ERK5 icKO mice in their sniffing duration during the second exposure (p = 0.11). This suggests that the 30-min olfactory memory for ERK5 icKO mice was comparable to control mice. Control mice maintained a similar degree of memory at 240 min and 300 min as it did at 30 min. However, ERK5 icKO mice did not exhibit statistically significant olfaction memory from 240 min onward. Thus, the short term olfactory memory of ERK5 icKO mice decayed much faster than that of the control mice, suggesting that they have impaired short-term olfactory memory.

### Reduced Detection Sensitivity to Chemically Defined Odorants and Pheromones in ERK5 icKO Mice

We next examined the sensitivity of odor detection using an olfactory threshold assay. ERK5 icKO and control mice were presented with pairs of cotton swabs laced with mineral oil or increasing concentrations of 1-octanol (1–500 µM). Although both groups of mice detected 500 µM 1-octanol equally well, ERK5 icKO mice failed to detect 1-octanol at 100 µM while control mice clearly did (Fig. 5A; *t*-test, *p* = 0.008). Similarly, control mice detected 2-heptanone, a mouse pheromone [27]–[29] at lower concentrations than ERK5 icKO mice did, and there was a statistically significant difference between the two animals at 100 µM 2-heptanone (Fig. 5B; *t*-test, *p* = 0.004).

We also examined the sensitivity of ERK5 icKO mice to farnesene, a synthetic, aggression-evoking pheromone [30], [31]. ERK5 icKO mice were only able to detect farnesene at the highest concentration tested (500 µM) while control mice detected it at 100 µM (Fig. 6A; *t*-test, *p* = 0.009). Furthermore, mice were also examined for their detection of TMT, a component of fox scent that is known to elicit innate fear and alarm in rodents [24], [32], [33]. When mice detect TMT, they will typically avoid it and may exhibit freezing behavior. Control mice avoided cotton swabs laced with TMT at 10 and 50 µM (Fig. 6B) while ERK5 icKO did not (*t*-test_10 µM_, *p* = 0.04; *t*-test_50 µM_, *p* = 0.05). It is possible that ERK5 icKO mice have reduced sensitivity to detect TMT at 10 and 50 µM. Alternatively, ERK5 icKO mice may have reduced intrinsic innate fear of TMT and therefore do not avoid the TMT-laced cotton swab as much as control mice. To distinguish between these possibilities, we used a separate cohort of mice and a 3-chamber apparatus to directly test their intrinsic innate fear of TMT at a higher concentration. Mice were placed in the middle chamber, TMT (5 mM) and vehicle control were placed in the other two chambers, separately. The duration of investigation toward each chamber and the percent time mice froze were quantified. Although both control and ERK5 icKO mice froze some of the time and spent less time investigating the chamber associated with TMT, ERK5 icKO mice clearly avoided TMT even more so than control mice (Fig. 6C; *t*-test_Control_, *p* = 0.002; *t*-test_ERK5 icKO_, *p*<0.001). They also froze more and moved less than control mice (Fig. 6D; *t*-test_Freeze_, *p* = 0.05; *t*-test_Move_, *p* = 0.05). Thus, ERK5 icKO mice do not exhibit reduced intrinsic innate fear when a sufficient concentration of TMT was present; if anything, their innate fear is enhanced. Together, our data suggest that ERK5 icKO mice have reduced sensitivity to detect odorants and pheromones at lower concentrations. However, *erk5* deletion and subsequent reduction of adult neurogenesis do not impair innate fear.

### ERK5 icKO Mice do not Prefer Mouse Urine Collected from Normal Females to that from Ovariectomized Females

Since ERK5 icKO mice displayed deficits in detecting lower concentrations of chemically defined odorants and pheromones, we next evaluated their responses to various mouse urine samples, which contain complex mixtures of odorants and pheromones. We first performed the olfactory habituation/dishabituation assay with mouse urine (1∶50 dilution) and found no distinguishable differences between control and ERK5 icKO mice in their ability to discriminate between various urine samples (Fig. 7A; ANOVA_vehicle_, *p* = 0.08; ANOVA_normal female urine_, *p* = 0.99; ANOVA_ovariectomized female urine_, *p* = 0.16; ANOVA_male urine_, *p* = 0.14; ANOVA_pregnant female urine_, *p* = 0.39; ANOVA_lactating female urine_, *p* = 0.08). Next we assessed if any overall deficits existed between groups of mice in the urine preference assay using different cohorts of mice. ERK5 icKO and control mice were presented with two cotton swabs laced with a pair of undiluted urine samples collected from groups of adult mice; their sniffing duration toward each cotton swab was recorded and quantified. Both male control and male ERK5 icKO mice showed a preference for female mouse urine relative to male mouse urine; there was no difference between the two genotypes (Fig. 7B; *t*-test, *p* = 0.68). Neither group of mice showed any preference between lactating and pregnant female mouse urine (Fig. 7C; *t*-test_Control_, *p* = 0.13; *t*-test_ERK5 icKO_, *p* = 0.76). Interestingly, however, although control mice preferred urine collected from normal female mice over that from ovariectomized females, ERK5 icKO mice showed no such preference (Fig. 7D; *t*-test_Control_, *p* = 0.003; *t*-test_ERK5 icKO_, *p* = 0.50).

### ERK5 icKO Mice are Impaired in Odor-cued Associative Olfactory Learning

To examine if conditional deletion of *erk5* and subsequent impairment of adult neurogenesis adversely affects acquisition of odor-cued associative olfactory tasks, mice were trained in a sand-digging based associative olfactory discrimination assay as described previously [20]. Mice were presented with two sand dishes, each laced with mouse urine (1∶50 dilution) from pregnant females or lactating females, respectively. A food reward was buried in the sand at the bottom of the dish laced with pregnant female urine. Thus, animals had to use olfactory cues to discriminate between the two urine samples to retrieve the food reward. This type of odor-cued associative task is hippocampus-independent [34]–[37]. Over the course of 5 training days, control mice learned that the food reward was associated with pregnant female mouse urine but not with lactating female mouse urine; learning was evidenced by the increase in correct choices (Fig. 8A). However, ERK5 icKO mice showed no such learning and there was a statistically significant difference between the two groups of mice (ANOVA, *p* = 0.03).

**Table 1:** List of odorants used in behavior assays.

Assay	Odorant	Concentration
Olfactory Habituation	Citralva	1 mM
	Isoamyl Acetate (IAA)	50 µM
	Ethyl Vanillin	50 µM
	Adult Female Mouse Urine	1∶50 Dilution
	Ovariectomized Female Mouse Urine	1∶50 Dilution
	Adult Male Mouse Urine	1∶50 Dilution
	Pregnant Female Mouse Urine	1∶50 Dilution
	Lactating Female Mouse Urine	1∶50 Dilution
Olfactory Memory	S-Terpinen-4-ol	1 mM
	Acetophenone	1 mM
	1-Octanol	1 mM
	Ethyl Vanillin	1 mM
Olfactory Threshold	1-Octanol	1 µM –500 µM
	2-Heptanone	1 µM –500 µM
	Farnesene	1 µM –500 µM
	2,3,5-Trimethyl-3-thiazoline (TMT)	1 µM –50 µM
Innate Fear	2,3,5-Trimethyl-3-thiazoline (TMT)	5 mM
Olfactory Preference	Male Mouse Urine	10 µL Undiluted
	Adult Female Mouse Urine	10 µL Undiluted
	Pregnant Female Mouse Urine	10 µL Undiluted
	Lactating Female Mouse Urine	10 µL Undiluted
	Adult Female Mouse Urine	10 µL Undiluted
	Adult Ovariectomized Female Mouse Urine	10 µL Undiluted
Odor-cued Associative Olfactory Learning	Pregnant Female Mouse Urine	1∶50 Dilution
	Lactating Female Mouse Urine	1∶50 Dilution
	Adult Female Mouse Urine	1∶50 Dilution
	Adult Ovariectomized Female Mouse Urine	1∶50 Dilution

This defect in odor-cued associative olfactory learning was confirmed with another pair of urine samples: normal adult female mouse urine vs. ovariectomized female mouse urine (Fig. 8B). Since control mice had a preference for normal adult female mouse urine over ovariectomized female mouse urine, we paired the food reward with ovariectomized female mouse urine. Over the course of a 6-day training paradigm, control mice learned significantly better than ERK5 icKO mice to discriminate between the urine pair and to associate ovariectomized female mouse urine with the food reward (ANOVA, *p* = 0.01). These data suggest that ERK5 icKO mice are impaired in the acquisition of odor-cued associative olfactory learning.

## Discussion

The adult brain has the remarkable capacity to continuously generate new neurons in the SVZ and OB. However, the functional impact of these adult-born neurons on olfactory behavior is still elusive. Furthermore, signaling mechanisms regulating adult neurogenesis in the SVZ/OB are not well defined. The objective of this study was to investigate the role of ERK5 MAP kinase in the regulation of adult SVZ/OB neurogenesis and associated olfactory behavior.

Although several studies have attempted to address the role of adult neurogenesis in olfactory behavior, findings from different studies are contradictory and the physiological function of adult-born neurons in the OB is still unclear [6], [9], [38], [39]. For example, genetic expression of a lethal diphtheria toxin fragment (DTA) in adult-born neurons, which kills these neurons, led to a substantial loss of adult-born granule cells in the OB and a much smaller OB [40]. Surprisingly, despite these structural changes in the OB, no deficits in olfactory behavior were observed, casting doubt as to whether adult-born neurons in the OB influence olfactory behavior. However, a subsequent study using lateral ventricle infusion of AraC to ablate adult neurogenesis in the SVZ/OB in mice showed that a reduction in OB adult neurogenesis reduces odor detection sensitivity and impairs short-term (hours) olfactory memory but does not affect odor discrimination or reward-associated long-term memory (days) [21]. Although results from this study generated much excitement in the field [9], [39], [41], they contradicted results from other reports [34], [42], [43]. For example, left ventricle infusion of AraC in another study implicated OB adult neurogenesis in long-term olfactory memory [34]. Furthermore, cranial irradiation of the SVZ, which robustly impaired adult neurogenesis in the OB, showed that only long-term olfactory memory, but not the threshold for odor detection or short-term olfactory memory, is affected [43]. In addition, NCAM knockout mice have diminished OB neurogenesis, and are deficient in the odor discrimination task while both the detection threshold for odors and short-term olfactory memory are unaltered [42]. Thus, the exact function of adult-born OB neurons is still highly debated.

Several factors may contribute to this controversy including the specificity of methods used to ablate or suppress adult neurogenesis. Although effective at suppressing adult neurogenesis, x-irradiation or anti-mitotic drugs are not specific for adult-born neurons. They target all dividing cells, may alter the neurovascular niche important for adult neurogenesis, and induce neural inflammation. The side effects intrinsic to these methods may be confounding factors contributing to inconsistent behavior results. Indeed, two studies using similar protocols for AraC administration to the lateral ventricle to suppress adult neurogenesis yielded opposite conclusions concerning a role for adult neurogenesis in long-term olfactory memory [21], [34]. Studies using traditional knockout of genes important for neurogenesis are also useful, but their interpretations are limited by widespread abnormalities of brain structure or compensatory effects elicited during development. Transgenic expression of a lethal gene, such as diphtheria toxin or thymidine kinase [40], [44], to kill adult-born neurons is more specific to adult neural stem/progenitor cells. However, large amounts of cell death in the SVZ-RMS-OB axis may interfere with normal olfactory function. These technical issues make it difficult to establish a definitive connection between adult neurogenesis and olfactory behavior.

The existing controversy necessitates studies using more specific genetic approaches with less off-target side effects to manipulate adult neurogenesis. Clearly, identification of a signaling molecule that is required for adult neurogenesis which is only expressed in neurogenic regions of the brain, coupled with a transgenic mouse engineered to temporally and spatially delete that gene only in adult neurogenic regions would be a powerful tool to evaluate the relationship between adult neurogenesis and olfaction. This study identifies *erk5* as such a candidate gene. Here we report that in the adult mouse brain, ERK5 MAP kinase is selectively expressed in the neurogenic regions including the SVZ, along the RMS, and in the center of the granular cell layer of the OB where adult-born neurons exit the RMS. Furthermore, conditional deletion of the *erk5* gene specifically in the neurogenic regions attenuated SVZ/OB neurogenesis *in vivo*. These data suggest an important role for ERK5 in the regulation of adult neurogenesis along the SVZ/RMS/OB axis.

We demonstrate that *erk5* deletion specifically in neurogenic regions of the adult mouse brain did not affect olfactory discrimination in the habituation/dishabituation assay. However, ERK5 icKO mice forget a previously exposed odor much faster and have reduced short-term olfactory memory compared with control littermates. Furthermore, ERK5 icKO mice exhibit reduced detection sensitivity to 1-octanol, a chemically defined odor, at a lower concentration. These results support the findings by Breton-Provencher et al. [21].

Although adult neurogenesis has been implicated in regulating pheromone-based animal behaviors in mice, such as mating, paternal recognition, and male-male aggression [33], [45], [46], it is not known whether adult neurogenesis influences the detection sensitivity of pheromones. We report here that ERK5 icKO mice exhibit reduced detection sensitivity to 2-heptanone, a mouse pheromone [27]–[29], to farnesene, a synthetic, aggression-evoking pheromone [30], [31], and to TMT, an innate fear- and alarm-inducing pheromone [24], [32], [33]. Furthermore, unlike control animals, ERK5 icKO male mice do not show preference to urine from normal over ovariectomized females. Following ovariectomy, circulating estradiol in rats is undetectable within 24 hours [47]. It is known that urine samples collected from ovariectomized female mice contain lower levels of protein and lipids than those from normal females [48]. Furthermore, certain fatty acids, including tridecanoic, palmitic and oleic acids, are present in the urine of normal but not ovariectomized female mice [48]. These decreases in circulating estrogen and biochemical constituents in the urine may cause and/or be an indicator of subtle differences in pheromones/odorants present in urine collected from ovariectomized vs. normal female animals. Together, these data suggest that adult neurogenesis in mice plays a critical role in their ability to detect pheromones in low concentrations. Since pheromones are likely present only in low abundance in their normal living environment, adult neurogenesis regulation of pheromone detection may underlie a variety of pheromone-based animal behaviors in mice.

Although several studies have evaluated the impact of adult neurogenesis on long-term memory retention (days) of odor-cued associative olfactory learning [10], [34], [43], adult neurogenesis has not been implicated in the acquisition of this learning task [34]. We demonstrate here that ERK5 icKO mice do not learn nearly as well as control mice to associate a specific mouse urine sample with a food reward. These data provide the first evidence that adult neurogenesis in the OB may be critical for the acquisition of odor-cued associative olfactory learning, a process that is independent of the hippocampus [34]–[37].

In summary, our data identify ERK5 MAP kinase as a novel signaling pathway regulating adult neurogenesis in the SVZ/OB. This is the first study utilizing genetic approaches to conditionally delete a specific gene selectively in the neurogenic regions of the adult brain to demonstrate a causal relationship between adult neurogenesis and several distinct forms of olfactory behavior, including detection sensitivity to odorants and pheromones, short-term olfactory memory, as well as acquisition of odor-cued associative olfactory learning.
