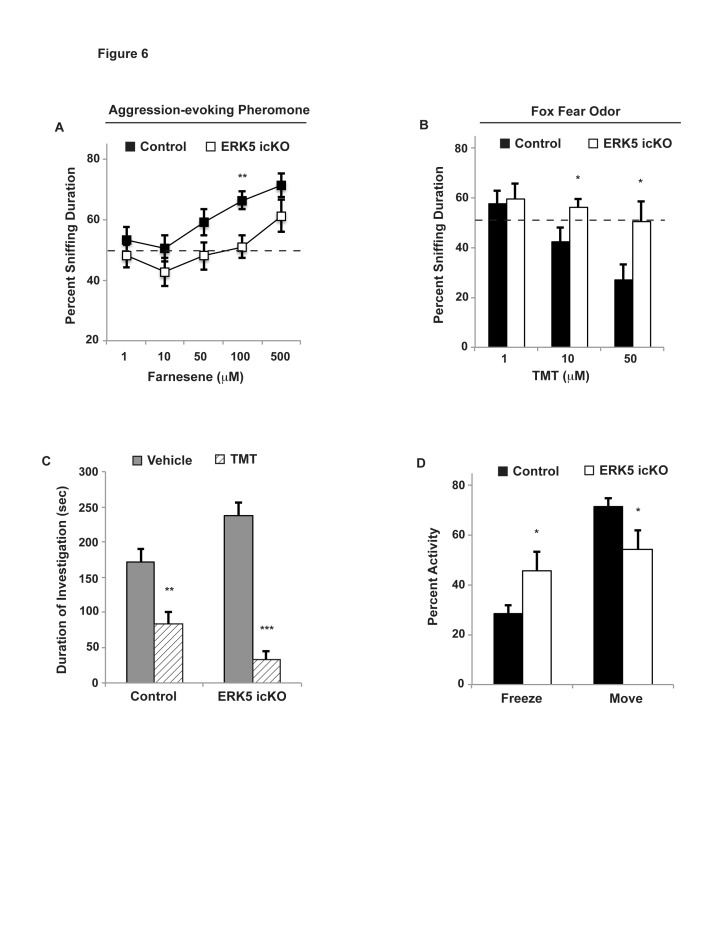# Correction: Inducible and Targeted Deletion of the ERK5 MAP Kinase in Adult Neurogenic Regions Impairs Adult Neurogenesis in the Olfactory Bulb and Several Forms of Olfactory Behavior

**DOI:** 10.1371/annotation/c8d48e05-6465-4b1c-963a-ccab25274fa7

**Published:** 2013-10-21

**Authors:** Yung-Wei Pan, Chay T. Kuo, Daniel R. Storm, Zhengui Xia

The legend of section D in Figure 6 was removed during the production process. Please see the corrected Table 6 here: 

**Figure:**